# Driving force of condomless sex after online intervention among Chinese men who have sex with men

**DOI:** 10.1186/s12889-019-7307-y

**Published:** 2019-07-22

**Authors:** Wenting Huang, Dan Wu, Stephen W. Pan, Katherine Li, Jason J. Ong, Hongyun Fu, Chuncheng Liu, Jessica Mao, Joseph D. Tucker, Weiming Tang

**Affiliations:** 1 SESH (Social Entrepreneurship to Spur Health) Team, Guangzhou, China; 2 University of North Carolina Project-China, Guangzhou, China; 3 Department of Public Health, Xi’an Jiaotong-Liverpool University, Suzhou, China; 4 Weill Cornell Medical College, New York, USA; 5 Faculty of Infectious and Tropical Disease, London School of Hygiene and Tropical Medicine, London, UK; 6 Department of Sociology, University of California, San Diego, La Jolla USA; 7 Department of Internal Medicine, Johns Hopkins Hospital, Baltimore, USA; 8 School of Medicine, University of North Carolina at Chapel Hill, Chapel Hill, USA; 9 Dermatology Hospital of Southern Medical University, Guangzhou, China

**Keywords:** HIV, MSM, Condom use

## Abstract

**Background:**

Condom use remains consistently low among Chinese men who have sex with men (MSM). This study aims to identify factors associated with condom use after online video intervention.

**Methods:**

This is a secondary data analysis of data collected from an online non-inferiority trial comparing the effectiveness of two condom use promotion video interventions among Chinese MSM. Participants from the two groups were combined since the effectiveness of two video interventions were shown to be non-inferior. Univariable and multivariable logistic regression were used to identify factors associated with condomless sex after the intervention during the follow-up interval.

**Results:**

Overall, 1173 participants were recruited at baseline and 791 (67.4%) completed the three-month follow-up survey. 57.3% (453/791) of the participants reported condomless sex after intervention in the three-month follow-up interval. MSM who have had sex under the influence of alcohol in the last 3 months (Odds Ratio(OR) = 1.90; 95% CI: 1.22, 2.97; Adjusted OR(AOR) = 1.79; 95% CI: 1.13, 2.83) and ever have had sex tourism (OR = 2.75; 95% CI: 1.34, 5.63; AOR = 2.40; 95% CI: 1.15, 5.07) at baseline were more likely to have condomless sex after intervention in the three-month follow-up period. MSM who had a higher level of community engagement in sexual health (OR = 0.54; 95% CI: 0.35, 0.82; AOR = 0.49; 95% CI: 0.32, 0.75 with substantial engagement) and who viewed additional condom promotion videos during the follow-up period by themselves (OR = 0.67; 95% CI = 0.50, 0.89; AOR = 0.67; 95% CI: 0.50, 0.91). were less likely to have condomless sex during the follow-up period.

**Conclusion:**

The intervention appeared to be effective among MSM who reported viewing additional condom promotion videos by themselves and more community engagement after the intervention. In MSM who reported risky sexual behaviors at baseline, the intervention appeared less effective. Tailored intervention videos that target particular subgroups, active in-person community engagement, and optimized intervention frequency should be considered in future sexual health interventions.

**Electronic supplementary material:**

The online version of this article (10.1186/s12889-019-7307-y) contains supplementary material, which is available to authorized users.

## Background

Although the national HIV prevalence has remained low (< 0.1%) in China, this prevalence has been increasing among men who have sex with men (MSM) [1]. HIV prevalence among MSM increased from 1.5% in 2005 to 8.0% in 2015 [2, 3]. It is estimated that 1 out of 70 men could be MSM. If the current HIV epidemic in MSM were not contained, the overall HIV prevalence would increase from 9.2% in 2016 to 12.6% in 2020 and 16.2% in 2025, [1]. Condom use is considered to be one of the most effective intervention strategies [4]. However, the rate of condom use remains consistently low among Chinese MSM. Previous systematic reviews found that the proportion of MSM who reported consistent condom use with male partners in the last 6 months ranged from 32.5 to 48.8% [2, 3]. In addition, one study found that condomless sex practices with female partners were common in bisexual MSM [5], which could act as a bridge in HIV transmission between male and female partners.

Recent studies suggest that the venues through which MSM seek sexual partners have shifted from offline to online [6]. Although seeking partners online may increase the risk of condomless sex [7], these online platforms could also provide a unique opportunity to disseminate intervention messages across regions and populations [8, 9]. Online platforms allow users to receive and forward text messaging, images, and videos. A meta-analysis showed that online interventions could effectively promote HIV testing among MSM in a wide range of settings [10]. Studies also suggested that online interventions can reduce risky sexual behaviors in MSM [9]. However, online interventions still facing many challenges like privacy concerns, limited breadth and depth of social media interaction, low “dosage” of the intervention, limited long-term effectiveness, etc. [9, 11].

We have conducted a non-inferiority randomized controlled trial using online video intervention to promote condom use among Chinese MSM [12]. The two video interventions in the study was found that could both effectively promote condom use in the follow-up period [12]. This study aimed to identify the factors that may impact the effectiveness of the online intervention in reducing condomless sex among Chinese MSM.

## Methods

### Study design and participants

This study is a secondary analysis of a non-inferiority randomized controlled trial on MSM in China conducted in 2015. The trial compared the effectiveness of a crowdsourced video to a social marketing video in promoting condom use among 1173 MSM who had condomless sex in the last 3 months in China [12]. The procedures of the trial including intervention and data collection, and have been previously described in detail [13]. Briefly, participants were recruited online through banner advertisements on Danlan’s online platforms, including danlan.org (a popular website for gay men), Blued (a popular gay dating mobile application in China), and Danlan’s social media platforms (Wechat and Weibo). The inclusion criteria were: 1) born biologically male; 2) ever had anal sex with a man; 3) 16 years old or older, and 4) had condomless sex in the last 3 months. Eligible participants were required to view a one-minute online video either developed through crowdsourcing contest or a social marketing company at baseline survey and then completed follow-up surveys at 3 weeks and 3 months after baseline. The effect of viewing the videos on reducing condomless sex was assessed by comparing the rate of condomless sex across three different periods: in the last three-month at baseline (100% as required by the eligible criteria), at the three-week follow-up (the rate of condomless sex during the last 3 weeks), and at the three-month follow-up (the rate of condomless sex during the last 3 months). Response rate and sociodemographic characteristics were comparable in both arms. Participants who completed the follow-up survey at 3 months were similar to those who did not (Additional file 1). In both the crowdsourced and social-marketing arms, the self-reported condomless sex in the last 3 months decreased significantly to 52.1% (t = − 8.46, *p* < 0.01) and 49.6% (t = − 8.78, *p* < 0.01) at three-month follow-up. The two videos were found to be comparable on reducing condomless sex (estimated difference: + 1.3%, 95% CI: − 4.8%, 7.4%) among MSM [12]. Therefore, we combined the data from the two arms in this secondary analysis. The original videos are available on Tencent Video (Shenzhen, Guangdong, China) at https://www.youtube.com/watch?v=Ib_7u5VHlCk and https://www.youtube.com/watch?v=WNtol1MdB0c.

Written informed consent was obtained online before the baseline survey from the eligible participants. Ethical approval was obtained from the institutional review boards at Guangdong Provincial Centre for Skin Diseases and STI Control, the University of North Carolina at Chapel Hill and the University of California, San Francisco, No. 15–1522.

### Measurement

The study only included surveys administered at baseline and 3 months after baseline using Qualtrics (Provo, Utah, USA) (Additional file 2). Baseline characteristics examined in this study included sociodemographic characteristics (age, education, income, student status, marital status, province of residence), sexual identity, HIV/syphilis testing history and HIV status, and sexual behavior (sex under the influence of alcohol, group sex, sex tourism, receptive anal sex with stable/casual male partners). Sex tourism was defined as having traveled outside of the city of residence to purchase sex with gifts or money [14]. Community engagement in sexual health was assessed at baseline. It was defined as awareness, participation, and advocacy of sexual health among community members. It was measured using a six-item scale developed and validated for MSM in China [15]. The six items are as follows: ever discussed HIV or other sexually transmitted infections (STI) testing or sexual health online; awareness of ongoing MSM sexual health events; ever encouraged others to get HIV/STI tested; ever accompanied others to a HIV/STI testing facility, ever helped organize a MSM sexual health campaign; and ever volunteered to help provide MSM sexual health services [15]. These community engagement items were categorized into none, minimal, moderate, and substantial engagement, according to a previous study [15]. In the three-month follow-up survey, participants were asked whether they had viewed any additional condom use promotion videos during the follow-up period by themselves.

In this secondary analysis, the outcome variable of condomless sex was defined as whether the participant had any anal sex or vaginal sex without a condom after intervention in the three-month follow-up period. Participants were asked whether they had any anal sex without a condom or any vaginal sex without a condom after intervention in the three-week follow-up period. They were also asked whether they had any anal sex without a condom or any vaginal sex without a condom after intervention in the three-month follow-up period. Because the three-month follow-up period included the three-week follow-up period, we also classified the participant as having had condomless sex in the last 3 months if he reported having had condomless sex in the three-week follow-up survey (Additional file 2).

### Data analysis

We used descriptive statistics to summarize sociodemographic characteristics and self-report of condomless sex after intervention in the three-month follow-up period. Chi-square tests were used for the bivariate analysis of the association between condomless sex and potential factors at baseline and 3 months after the intervention. Multivariable logistic regressions were used to identify further the association between condomless sex and the potential factors when adjusted for age, education, income, marital status, and viewing either the crowdsourced video or the social marketing video. Statistical analyses were conducted using Stata/SE 13.0 (StataCorp, Texas, USA). The estimated effect sizes were reported as odds ratios (OR) with 95% confidence intervals (CIs) and *p* values. Statistical significance was based on *p* value < 0.05.

## Results

We recruited 1173 MSM who had condomless sex in the last 3 months at baseline. 791 (67.4% of baseline) participants completed the three-month follow-up. Table 1 presents the sociodemographic characteristic of the overall sample. The average age of men was 25 years old (SD 6.7) and most participants self-identified as gay (72.1%). The majority had completed college-level education or above (68.0%), were not students (63.7%), and have never married (84.3%). Men were recruited from all of China’s regions, with the greatest proportion (335, 28.6%) recruited from the north (Beijing, Tianjing, Hebei, Shanxi, Neimenggu, and Henan province) and fewer (97, 8.3%) from the northeast (Heilongjiang, Jilin, and Liaoning). The socio-demographic and sexual behavior characters were similar between those who completed the 3 months follow-up survey and those who did not (Additional file 2).

**Table 1:** Sociodemographics of Chinese MSM retained at three-month follow up, 2015 (*n* = 791)

	All N (%)
Age^a^ (mean, SD)	25, 6.7
Gender Identity	
Gay	570 (72.1)
Others^b^	221 (27.9)
Education	
High School or Below	253 (32.0)
College or above	538 (68.0)
Annual income, US$	
< 2700	236 (29.8)
2701–5500	206 (26.0)
5501–9100	206 (26.0)
9101–15000	95 (12.0)
> 15001	48 (6.1)
Student status	
No	496 (62.7)
Yes	295 (37.3)
Marital status	
Never married	667 (84.3)
Ever married	124 (15.7)
Region^c^	
Northeast	64 (8.0)
North	222 (28.1)
Northwest	115 (14.5)
Southwest	122 (15.4)
South	107 (13.5)
East	161 (20.4)

^a^3 missing values

^b^Including bisexual, heterosexual, and unsure

^c^Northeast: Heilongjiang, Jilin, and Liaoning; North: Beijing, Tianjin, Hebei, Shanxi, Inner Mongolia, and Henan; Northwest: Shaanxi, Gansu, Ningxia, Xinjiang, and Qinghai; Southwest: Chongqing, Sichuan, Guizhou, Yunnan, Tibet, and Guangxi; South: Guangdong, Hubei, Hunan, Hong Kong, Macao, and Hainan; East: Shanghai, Jiangsu, Zhejiang, Shandong, Anhui, Jiangxi, Fujian, and Taiwan

^ƚ^*p*-value for t-test

Overall, 57.3% (453/791) of the participants reported condomless sex after intervention in the three-month interval. Compared with participants who did not have condomless sex, MSM who had condomless sex were on average older (mean age of 26, SD 6.3 vs. 24, SD 7.0). Of the MSM who reported having had condomless sex, fewer self-identified as gay (68.7% vs. 76.7%), fewer self-reported as students (33.8% vs. 42.0%) and fewer were married (81.0% vs. 88.8%) compared with participants who did not have condomless sex. These two groups of participants were similar in education level and geographic region (Table 2).

**Table 2:** Comparison of baseline sociodemographics and behavior characteristics between Chinese MSM who had condomless sex (*n* = 453) and those who did not (*n* = 338) during the three-month follow-up period, 2015

	Condomless sex, N (%)		*χ* ^2^	X*P* value
	No	Yes		
Age^a^ (mean, SD)	24, 6.3	26, 7.0	−3.23^†^	< 0.01^‡^
Gender Identity				
Gay	259 (76.7)	311 (68.7)	6.11	0.01
Others^b^	79 (23.4)	142 (31.4)		
Education				
High School or Below	111 (32.8)	142 (31.4)	0.20	0.66
College or above	227 (67.2)	311 (68.7)		
Annual income, US$				
< 2700	134 (39.6)	102 (22.5)	33.83	< 0.01
2701–5500	74 (21.9)	132 (29.1)		
5501–9100	87 (25.7)	119 (26.3)		
9101–15000	32 (9.5)	63 (13.9)		
> 15001	11 (3.3)	37 (8.2)		
Student status				
No	196 (58.0)	300 (66.2)	5.62	0.02
Yes	142 (42.0)	153 (33.8)		
Marital status				
Never married	300 (88.8)	367 (81.0)	8.78	< 0.01
Ever married	38 (11.2)	86 (19.0)		
Ever tested for HIV/Syphilis				
No	157 (46.5)	187 (41.3)	2.10	0.15
Yes	181 (53.6)	266 (58.7)		
HIV status				
Positive	13 (3.9)	12 (2.7)	2.19	0.34
Negative	159 (47.0)	234 (51.7)		
Unknown	166 (49.1)	207 (45.7)		
Sex under the influence of alcohol ^c^				
No	307 (90.8)	380 (83.9)	8.17	< 0.01
Yes	31 (9.2)	73 (16.1)		
Group sex^d^				
No	308 (91.1)	400 (88.3)	1.64	0.20
Yes	30 (8.9)	53 (11.7)		
Ever had sex tourism				
No	328 (97.0)	418 (92.3)	8.20	< 0.01
Yes	10 (3.0)	35 (7.7)		
Receptive anal position with stable male partner^c^				
No	215 (63.6)	232 (51.2)	12.10	< 0.01
Yes	123 (36.4)	221 (48.8)		
Receptive anal position with casual male partner^c^				
No	161 (47.6)	176 (38.9)	6.10	0.01
Yes	177 (52.4)	277 (61.2)		
Viewed additional condom use promotion videos^ce^				
No	179 (53.0)	284 (62.7)	7.56	< 0.01
Yes	159 (47.0)	169 (37.3)		
Community engagement in sexual health				
None/Minimal	76 (22.5)	136 (30.0)	8.47	0.01
Moderate	182 (53.9)	240 (53.0)		
Substantial	80 (23.7)	77 (17.0)		

^a^3 missing values

^b^Including bisexual, heterosexual, and unsure

^c^in the last 3 months

^d^ in the last 12 months

^e^at the 3-month follow up survey

^†^*t*-value for t-test

^‡^*p*-value for t-test

In terms of sexual behaviors, a higher proportion of MSM who engaged in condomless sex reported that they had sex under the influence of alcohol in the past 3 months (16.1%), had sex tourism (7.7%) and had receptive anal sex at baseline (48.8% with a stable partner, and 61.2% with a casual partner), compared to those who consistently used condom during the follow-up interval. In addition, MSM who had condomless sex after intervention were less likely to view additional condom promotion videos during the three-month follow-up period by themselves (37.3%) and less likely to have a substantial level of community engagement (17.0%) than those who did not report condomless sex after the intervention. (Table 2).

The multivariable logistic regression models in Table 3 explores factors associated with having condomless sex in the 3 months after the intervention. The results showed that condomless sex after intervention was associated with following sexual risky behaviors at baseline: having had sex under the influence of alcohol in the last 3 months (AOR = 1.79, 95% CI: 1.13, 2.83) and ever having had sex tourism (AOR = 2.40, 95% CI: 1.15, 5.07). Engagement in receptive anal sex both with stable male partners (AOR = 1.84, 95% CI: 1.36, 2.50) and casual male partners at baseline (AOR = 1.48, 95% CI: 1.10, 1.99) were associated with high odds of condomless sex after the intervention. On the contrary, higher levels of community engagement in sexual health at baseline (AOR = 0.66, 95% CI: 0.46, 0.94 with moderate engagement; AOR = 0.49, 95% CI: 0.32, 0.75 with substantial engagement) and viewing additional condom promotion videos by themselves in the follow-up interval (AOR = 0.67, 95% CI: 0.50, 0.91) were significantly associated with low odds of having condomless sex after intervention during the follow-up period. Neither HIV/syphilis testing nor HIV status was significantly associated with condomless sex in the follow-up period (Table 3).

**Table 3:** Factors associated with condomless sex in the three-month follow-up period among Chinese MSM who participated in an online video intervention, 2015 (*n* = 791)

	Condomless sex OR (95% CI)	Condomless sex^a^ aOR (95% CI)
Gender Identity		
Gay	*ref*	*ref*
Others^b^	1.50 (1.09, 2.06)*	1.40 (1.00, 1.95)*
Student status		
No	*ref*	*ref*
Yes	0.70 (0.53, 0.94)*	1.47 (0.95, 2.27)
Ever tested for HIV/Syphilis		
No	*ref*	*ref*
Yes	1.23 (0.93, 1.64)	1.07 (0.79, 1.44)
HIV status		
Positive	*ref*	*ref*
Negative	1.59 (0.71, 3.58)	1.64 (0.72, 3.75)
Unknown	1.35 (0.60, 3.04)	1.56 (0.68, 3.57)
Sex under the influence of alcohol^c^		
No	*ref*	*ref*
Yes	1.90 (1.22, 2.97)*	1.79 (1.13, 2.83)*
Group sex^d^		
No	*ref*	*ref*
Yes	1.37 (0.85, 2.18)	1.22 (0.75, 2.00)
Ever had sex tourism		
No	*ref*	*ref*
Yes	2.75 (1.34, 5.63)*	2.40 (1.14, 5.03)*
Receptive anal position with stable male partner^c^		
No	*ref*	*ref*
Yes	1.67 (1.25, 2.22)*	1.85 (1.37, 2.50)*
Receptive anal position with casual male partner^c^		
No	*ref*	*ref*
Yes	1.43 (1.08, 1.90)*	1.47 (1.09, 1.97)*
Viewed additional condom use promotion videos^ce^		
No	*ref*	*ref*
Yes	0.67 (0.50, 0.89)*	0.67 (0.50, 0.91)*
Community engagement in sexual health		
None/ Minimal	*ref*	*ref*
Minimal	0.74 (0.52, 1.36)	0.66 (0.46, 0.94)*
Substantial	0.54 (0.35, 0.82)*	0.49 (0.32, 0.75)*

^a^Adjusted for age (3 missing values, continuous), education, income, marital status, and types of intervention videos. *N* = 788

^b^Including bisexual, heterosexual, and unsure

^c^in the last 3 months

^d^in the last 12 months

^e^collected at the 3-month follow up survey

*p-value< 0.05

## Discussion

Consistent condom use is still recommended for all key populations and adolescences from these key populations, including MSM, people in prison, sex workers, and transgender people. Previous studies reported that online interventions might be effective in changing behaviors like condom use [16] while some studies indicated that the effects on improving condom use were not significant [8, 17]. In this study, after the intervention, 57.3% of the participants still engaged in condomless sex after the intervention. This analysis extends the existing literature by identifying subgroups of MSM in whom the online intervention appeared to be effective.

We found that risky sexual behavior at baseline, including sex under the influence of alcohol or had sex tourism were associated with condomless sex after the intervention during the follow-up period. Previous studies suggested that people with some individual traits like risk-taking, sensation seeking, or sexual compulsivity might be more likely to engage in both alcohol use and risky sexual behaviors [18–20]. In the subgroups of MSM with these personal traits, the effectiveness of the online video intervention targeting on general MSM could be attenuated. Thus, when designing an online intervention, populations with such personal traits should be taken into special consideration. For example, novel, emotionally intense, fast-paced, and suspenseful videos may be more attractive to the risk-taking and sensation seeking population [20–22].

We also found that viewing additional condom promotion videos by themselves during the follow-up period was associated with condom use during the 3 months after the intervention. While the online condom intervention video may spur men to watch more sexual health relevant videos, viewing additional videos could potentially strengthen the effectiveness of the online video intervention. Also, additional condom promotion videos may serve as increasing dosing of the intervention. As previous a study indicated, the likelihood of consistent use condom increased with increasing exposure to intervention videos [23]. Increasing the dosage of videos intervention with similar concepts may not only reinforce the participant’s memory but also prolong the effect duration. This may help foster and sustain behavioral change. Exposing the participants to the same concept in multiple formats instead of one-off exposure could be considered in future behavioral change interventions.

The study finding suggests that community engagement in sexual health was associated with condom use, which is consistent with previous studies [24, 25]. Community engagement has been recognized as an important contributor to behavioral change [15, 26]. Rather than using community engagement as a dichotomous variable, in this study, we evaluated different levels of community engagement. Compared with minimal engagement in online and non-interactive events, moderate and substantial community engagement in active and in-person events may be more effective in facilitating consistent condom use. Therefore, active, in-person community events should be encouraged when incorporating community engagement as a component in future condom use interventions. An optimal way to increase community engagement for MSM would be the collaboration between the public health sector and local community-based organizations.

This study has several limitations. First, only 68% of participants were retained in the study at 3 months follow-up. Reasons for lost to follow-up were not collected in this study. However, the socio-demographic and sexual behavioral characteristics were similar between those who completed the study and those who did not, and the bias due to loss to follow-up should be minimal. Second, the participants in this study are relatively young. Young men may find online video interventions more engaging than older men. Third, characteristics like sociodemographic characteristics, having sex under the influence of alcohol, sex tourism, and receptive anal sex position, were only collected at baseline.

Given that the follow-up period was 3 months after the intervention, these characteristics were unlikely to change in such a short period. Fourth, all results in the survey were self-reported, so reporting bias should be considered. However, the survey was distributed online and completed anonymously, and such bias should be minimal [27]. Finally, this study followed 791 MSM only for 3 months. The findings in this study should be considered to be careful when generalizing to a larger MSM population and for longer durations.

## Conclusion

Having sex under the influence of alcohol, ever having engaged in sex tourism, and being engaged in receptive anal sex at baseline may attenuate the effectiveness of online condom promotion video interventions. Viewing additional condom use promotion videos and having a higher level of community engagement at baseline could strengthen the effectiveness of the intervention. Future sexual health interventions for MSM should consider tailoring intervention videos for particular MSM subgroups, incorporating active and in-person community engagement, and increasing the dosing of intervention materials.

## Additional files

Additional file 1: Comparison of participant baseline characteristics who retained (*N* = 791) and were not retained (*N* = 382) in the three-month study, China, 2015. (DOCX 17 kb)

Additional file 2: Questionnaire of the online survey conducted among Chinese MSM in 2015. (DOCX 78 kb)

## Data Availability

The datasets used and/or analyzed during the current study are available from the corresponding author on reasonable request.

## Abbreviations

CI: Confidence interval
MSM: Men who have sex with men
OR: Odds ratio
STI: Sexually transmitted infections
