# Supplementary material for: Driving force of condomless sex after online intervention among Chinese men who have sex with men
Source: BMC Public Health. 2019 Jul 22;19:978. doi: 10.1186/s12889-019-7307-y (PMC6647144; doi:10.1186/s12889-019-7307-y)
Supplement: Supplementary file 1 — Comparison of participant baseline characteristics who retained (N = 791) and were not retained (N = 382) in the three-month study, China, 2015. (DOCX 17 kb) [file 12889_2019_7307_MOESM1_ESM.docx]

**Supplement 1. Comparison of participant baseline characteristics who retained (N=791) and were not retained (N=382) in the three-month study, China, 2015**

|  | Retained  N (%) | Not Retained  N (%) | *P* value |
| --- | --- | --- | --- |
| Age ^a^ (mean, SD) | 25, 6.74 | 25, 6.88 | 0.51^ƚ^ |
| Gender identity |  |  |  |
| Gay | 570 (72.1) | 256 (67.0) | 0.08 |
| Others ^b^ | 221 (27.9) | 126 (33.0) |  |
| Education |  |  |  |
| High School or Below | 253 (32.0) | 135 (35.3) | 0.25 |
| College or above | 538 (68.0) | 247 (64.6) |  |
| Annual income, US$ |  |  |  |
| <2700 | 236 (29.9) | 91 (23.8) | 0.14 |
| 2701-5500 | 206 (26.0) | 103 (27.0) |  |
| 5501-9100 | 206 (26.0) | 122 (31.9) |  |
| 9101-15000 | 95 (12.0) | 42 (11.0) |  |
| >15001 | 48 (6.1) | 24 (6.3) |  |
| Student Status |  |  |  |
| No | 496 (62.7) | 251 (65.7) | 0.32 |
| Yes | 295 (37.3) | 131 (34.3) |  |
| Marital Status |  |  |  |
| Never married | 667 (84.3) | 310 (81.2) | 0.17 |
| Ever married | 124 (15.7) | 72 (18.9) |  |
| Region^c^ |  |  |  |
| Northeast | 64 (8.1) | 33 (8.6) | 0.99 |
| North | 222 (28.1) | 113 (29.6) |  |
| Northwest | 115 (14.5) | 52 (13.6) |  |
| Southwest | 122 (15.4) | 58 (15.2) |  |
| South | 107 (13.5) | 48 (12.6) |  |
| East | 161 (20.4) | 78 (20.4) |  |

^a^ 5 missing values: retained (n=788), not retained (n=380),

^b^ Including bisexual, heterosexual, and unsure

^c^ Northeast: Heilongjiang, Jilin, and Liaoning; North: Beijing, Tianjin, Hebei, Shanxi, Inner Mongolia, and Henan; Northwest: Shaanxi, Gansu, Ningxia, Xinjiang, and Qinghai; Southwest: Chongqing, Sichuan, Guizhou, Yunnan, Tibet, and Guangxi; South: Guangdong, Hubei, Hunan, Hong Kong, Macao, and Hainan; East: Shanghai, Jiangsu, Zhejiang, Shandong, Anhui, Jiangxi, Fujian, and Taiwan.

^ƚ^*P* value for t-test.
